# Subclinical inflammation on MRI of hand and foot of anticitrullinated peptide antibody–negative arthralgia patients at risk for rheumatoid arthritis

**DOI:** 10.1186/ar4536

**Published:** 2014-04-10

**Authors:** Hanna W van Steenbergen, Jessica AB van Nies, Tom WJ Huizinga, Monique Reijnierse, Annette HM van der Helm-van Mil

**Affiliations:** 1 Department of Rheumatology, Leiden University Medical Center, P.O. Box 9600, 2300 RC, Leiden, the Netherlands; 2 Department of Radiology, Leiden University Medical Center, P.O. Box 9600, 2300 RC, Leiden, the Netherlands

## Abstract

**Introduction:**

It is known that anticitrullinated peptide antibody (ACPA)–positive rheumatoid arthritis (RA) has a preclinical phase. Whether this phase is also present in ACPA-negative RA is unknown. To determine this, we studied ACPA-negative arthralgia patients who were considered prone to progress to RA for local subclinical inflammation observed on hand and foot magnetic resonance imaging (MRI) scans.

**Methods:**

We studied a total of 64 ACPA-negative patients without clinically detectable arthritis and with arthralgia of the small joints within the previous 1 year. Because of the character of the patients’ symptoms, the rheumatologists considered these patients to be prone to progress to RA. For comparisons, we evaluated 19 healthy, symptom-free controls and 20 ACPA-negative RA patients, who were identified according to the 1987 American Rheumatism Association criteria. All participants underwent MRI of unilateral wrist, metacarpophalangeal and metatarsophalangeal joints. Synovitis and bone marrow oedema (BME) were scored according to the OMERACT rheumatoid arthritis magnetic resonance imaging scoring system, and the scores were summed to yield the ‘MRI inflammation score’. Scores were compared between groups. Among the ACPA-negative arthralgia patients, MRI inflammation scores were related to C-reactive protein (CRP) levels and the tenderness of scanned joints.

**Results:**

MRI inflammation scores increased progressively among the groups of controls and ACPA-negative arthralgia and RA patients (median scores = 0, 1 and 10, respectively; *P* < 0.001). The MRI inflammation scores of ACPA-negative arthralgia patients were significantly higher than those of controls (*P* = 0.018). In particular, the synovitis scores were higher in ACPA-negative arthralgia patients (*P* = 0.046). Among the ACPA-negative arthralgia patients, inflammation was observed predominantly in the wrist (53%). The synovitis scores were associated with CRP levels (*P* = 0.007) and joint tenderness (*P* = 0.026). Despite the limited follow-up duration, five patients developed clinically detectable arthritis. These five patients had higher scores for MRI inflammation (*P* = 0.001), synovitis (*P* = 0.002) and BME (*P* = 0.003) compared to the other patients.

**Conclusion:**

Subclinical synovitis was observed in the small joints of ACPA-negative arthralgia patients, and especially in patients whose conditions progressed to clinically detectable arthritis. This finding suggests the presence of a preclinical phase in ACPA-negative RA. Further longitudinal studies of these lesions and patients are required to confirm this hypothesis.

## Introduction

Early recognition of rheumatoid arthritis (RA) and early treatment initiation of it have been proven to be effective in reducing the disease burden over time [1,2]. For the past few years, interest in the early disease phase has also covered the preclinical phase of RA [3]. It has been shown that RA-specific autoantibodies [4,5] and serologic inflammatory markers are increased months to years before development of RA [6,7]. Also, subclinical inflammation locally in the small joints of autoantibody-positive arthralgia patients without clinical arthritis was visualised using ultrasonography, positron emission tomography (PET) and magnetic resonance imaging (MRI) [8-10]. Previous studies that investigated the preclinical phase of RA mainly or solely focused on anticitrullinated peptide antibody (ACPA) positive RA. Consequently, it is not known whether ACPA-negative RA also has a preclinical phase. Nonetheless, up to half of all patients in early RA cohorts are ACPA-negative [1,11,12].

MRI is a suitable modality for studying early inflammatory changes in the small joints of patients in the preclinical phase of RA. It detects synovitis and is the only imaging modality that depicts bone marrow oedema (BME), an MRI feature that is strongly associated with disease progression [13-15]. The availability of dedicated MRI scanners have increased the accessibility and comfort of MRI scanning. Additionally, the presence of a validated scoring methodology (the Outcome Measures in Rheumatology Clinical Trials (OMERACT) rheumatoid arthritis magnetic resonance imaging scoring system (RAMRIS)) allows comparison of the extent and severity of MRI features for research purposes [15].

In the present study, we used MRI of the hand and foot to evaluate whether ACPA-negative RA, like ACPA-positive RA, has a preclinical phase with local inflammation in small joints. Persons with any type of arthralgia are prevalent in the general population and at rheumatologic outpatient clinics. Because the majority of arthralgia patients are ACPA-negative and will never develop RA, it is challenging to identify the ACPA-negative arthralgia patients that might be in a preclinical phase of RA. We studied ACPA-negative patients without clinical arthritis and with recent-onset arthralgia of small joints who, because of the character of their symptoms, were considered prone to have disease likely to progress to RA by the treating rheumatologists. For comparisons, healthy controls and ACPA-negative RA patients were also studied.

## Methods

### Participants

Three groups of participants were studied. The first group consisted of 64 ACPA-negative arthralgia patients recruited at the Leiden University Medical Center between April 2012 and June 2013. The rheumatologists were requested to include patients who presented to the outpatient clinic without clinical arthritis upon physical examination but with arthralgia of the hand or foot joints of less than 1 year’s duration of a type that was considered to have an increased chance to progress to RA. This suspicion was based on symptoms and signs, combined with the gut feelings of the rheumatologists. Hence, based on the rheumatologists’ clinical impression, these patients were considered to be in a preclinical phase of RA. The rheumatologists were encouraged to include patients whom they had otherwise also followed and not discharged because they were concerned that these patients had an increased risk for RA development. Because no type of arthralgia has yet been defined to be specific for the preclinical phase of RA, we could not assign more specific criteria with regard to the type of arthralgia patients to be included. Importantly, when another explanation for the patients’ arthralgia was more likely, such as fibromyalgia, osteoarthritis or an inflammatory rheumatic disease, these patients were not included. In our present study, among all patients with arthralgia, the 64 patients who tested negative for ACPA (anticyclic citrullinated peptide 2–negative) (Euro Diagnostica AB, Nijmegen, the Netherlands) were selected. The second group comprised 20 ACPA-negative patients who met the 1987 American Rheumatism Association criteria for RA [16]. These patients were included in the Leiden Early Arthritis Clinic cohort between August 2010 and July 2012. The third group consisted of 19 healthy controls without joint symptoms. Written informed consent was obtained from all participants. Approval of the study protocol was obtained from the local Medical Ethics Committee of the Leiden University Medical Center.

### Magnetic resonance imaging

All participants underwent MRI of the wrist, metacarpophalangeal (MCP) joints and metatarsophalangeal (MTP) joints with an ONI MSK Extreme 1.5 T MRI scanner (GE Healthcare Life Sciences, Madison, WI, USA). In the arthralgia and RA patients, MRI of the most painful side was performed within 2 weeks after the first visit. In cases of equally severe symptoms on both sides, the dominant side was scanned. Patients were asked not to use any nonsteroidal anti-inflammatory drugs (NSAIDs) during the 24 hours before undergoing MRI. The healthy symptom-free controls underwent MRI of the dominant side. The following sequences were acquired for MCP joints and wrists: a coronal T1-weighted fast spin echo (FSE) sequence, a coronal T2-weighted FSE sequence with fat saturation and, after intravenous gadolinium contrast enhancement (0.1 mmol/kg), coronal and axial T1-weighted FSE sequences with fat saturation. Axial T1-weighted FSE sequences and T2-weighted FSE sequences with fat saturation of MTP joints were acquired. Owing to time constraints, post-contrast-enhanced images were not obtained of the MTP joints. For ethical reasons, contrast agents were not administered in controls. Synovitis and BME were scored quantitatively according to the OMERACT RAMRIS system [15]. The sum of the synovitis and BME scores yielded the ‘MRI inflammation score’. Scoring was performed by one trained reader, 47% of the scans were read twice and the within-reader intraclass correlation coefficient for the MRI inflammation score was 0.91.

### Analyses

Comparisons were made using a Mann-Whitney *U* test, Kruskal-Wallis test or χ^2^ test as appropriate. In the ACPA-negative arthralgia patients, linear regression analyses were used to study whether C-reactive protein (CRP) level was associated with MRI-determined inflammation scores. The associations between tenderness and degree of inflammation observed on MRI scans were tested by performing generalized estimating equations. This model took into account that, in every patient, ten joints were assessed. The unstructured correlation matrix was used. SPSS version 20.0 software (SPSS, Chicago, IL, USA) was used for calculations. *P*-values <0.05 were considered significant.

## Results

### ACPA-negative arthralgia patients prioritized by the rheumatologists

The rheumatologists were requested to state the primary reasons why they assumed that the arthralgia patients had an increased risk for RA development. The main reasons provided were joint pain that was worst in the early morning and improved with movement during the day (thus making it an inflammatory type of arthralgia), the presence of morning stiffness of ≥60 minutes and/or a positive family history of RA. The baseline characteristics of the ACPA-negative arthralgia patients, as well as those of the ACPA-negative RA patients and symptom-free controls, are presented in Table 1. The ACPA-negative arthralgia patients who were considered at risk for progression to RA had a mean age of 42 years, and 72% were female. The symptoms of most patients had started gradually (75%) and initially involved the upper extremities (73%). Tender joints were localized predominantly in the proximal interphalangeal (PIP) joints (60%) and the MCP joints (52%). Nine patients (14%) were rheumatoid factor (RF)-positive.

**Table 1:** **Patient characteristics**^**a**^

**Characteristics**	**Symptom-free controls**	**ACPA-negative arthralgia**	**ACPA-negative RA**
	**(*n*** **= 19)**	**(*n*** **= 64)**	**(*n*** **= 20)**
Mean age, yr (SD)	46.2 (11.8)	41.9 (14.3)	58.7 (14.5)
Females, *n* (%)	15 (78.9)	46 (71.9)	11 (55.0)
Positive family history of RA, *n* (%)	N/A	25 (39.1)	4 (20.0)
Median symptom duration at time of inclusion, wk (IQR)	N/A	13.4 (8.4 to 26.4)	17.6 (11.5 to 25.9)
Gradual symptom onset, *n* (%)	N/A	48 (75.0)	12 (60.0)
Initial symptom localization, *n* (%)	N/A		
Upper extremities, *n* (%)		47 (73.4)	10 (50.0)
Lower extremities, *n* (%)		2 (3.1)	4 (20.0)
Upper and lower extremities, *n* (%)		15 (23.4)	6 (30.0)
Symmetrical localization, *n* (%)	N/A	46 (71.9)	13 (65.0)
Median morning stiffness, min (IQR)	N/A	45 (15 to 90)	120 (30 to 120)
Median tender joint count in 68 joints (IQR)	0	5.5 (3 to 10.8)	12 (4.8 to 17.8)
Median swollen joint count 66 joints (IQR)	0	0	6 (4 to 11)
ACPA positivity (>7.0 IU/ml), *n* (%)	N/A	0	0
IgM RF positivity (>3.5 IU/ml), *n* (%)	N/A	9 (14.1)	3 (15.0)
Increased CRP level (>10 mg/L), *n* (%)	N/A	10 (15.6)	11 (55.0)

^a^ACPA, Anticitrullinated peptide antibody; CRP, C-reactive protein; IQR, Interquartile range; IgM RF, Immunoglobulin M rheumatoid factor; N/A, Not applicable; RA, Rheumatoid arthritis; SD, Standard deviation.

### Magnetic resonance imaging findings in the three groups

**Figure 1:**
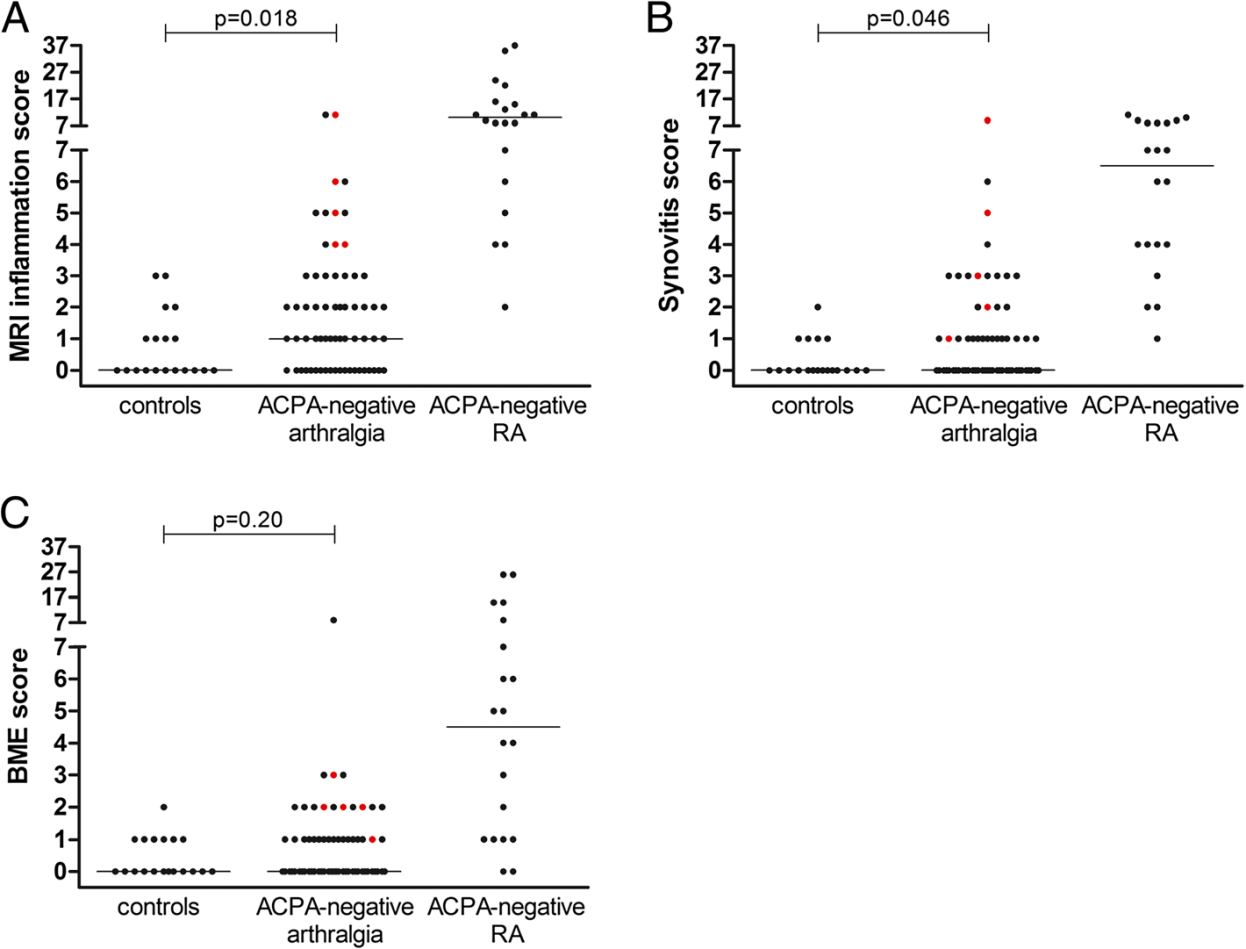
**Magnetic resonance imaging-based inflammation scores shown separately for the three study groups. (A)** Magnetic resonance imaging (MRI) inflammation scores (synovitis plus bone marrow edema (BME)). **(B)** Synovitis scores. **(C)** Bone marrow oedema scores. The three study groups are the symptom-free controls, the anticitrullinated peptide antibody (ACPA)–negative arthralgia patients and the ACPA-negative rheumatoid arthritis (RA) patients, based on the 1987 criteria for RA [16]. The scores presented are for all participants individually (dots) and the median scores per group (horizontal lines). The red dots indicate the ACPA-negative patients who developed clinically detectable arthritis during the median follow-up of 9 months. The *y*-axes are split because RA patients had higher scores than the symptom-free controls and ACPA-negative arthralgia patients. The presented *P*-values were obtained by comparing the scores of ACPA-negative arthralgia patients and symptom-free controls. All *P* < 0.001 for differences in MRI-based inflammation, synovitis and BME scores between the three groups.

The median (interquartile range (IQR)) MRI inflammation scores in symptom-free controls, ACPA-negative arthralgia patients and ACPA-negative RA patients were 0 (0 to 1), 1 (1 to 3) and 10 (10 to 16), respectively (*P* < 0.001) (Figure 1).

### Magnetic resonance imaging findings in ACPA-negative arthralgia patients and symptom-free controls

The ACPA-negative arthralgia patients were compared with the symptom-free controls (Figure 1). Eight (42.1%) of the nineteen symptom-free controls and forty-four (68.8%) of the sixty-four ACPA-negative arthralgia patients had any sign of inflammation based on MRI (inflammation score ≥1) (*P* = 0.035). The median MRI inflammation scores were significantly higher in the ACPA-negative arthralgia patients than in controls (*P* = 0.018). Subsequently, synovitis and BME scores were evaluated separately. This analysis showed that synovitis scores were significantly higher in ACPA-negative arthralgia patients than in controls (*P* = 0.046), in contrast to BME patients (*P* = 0.20) (Figure 1). Thus, compared to controls, patients with ACPA-negative arthralgia in particular had higher subclinical synovitis scores of small joints.

**Figure 2:**
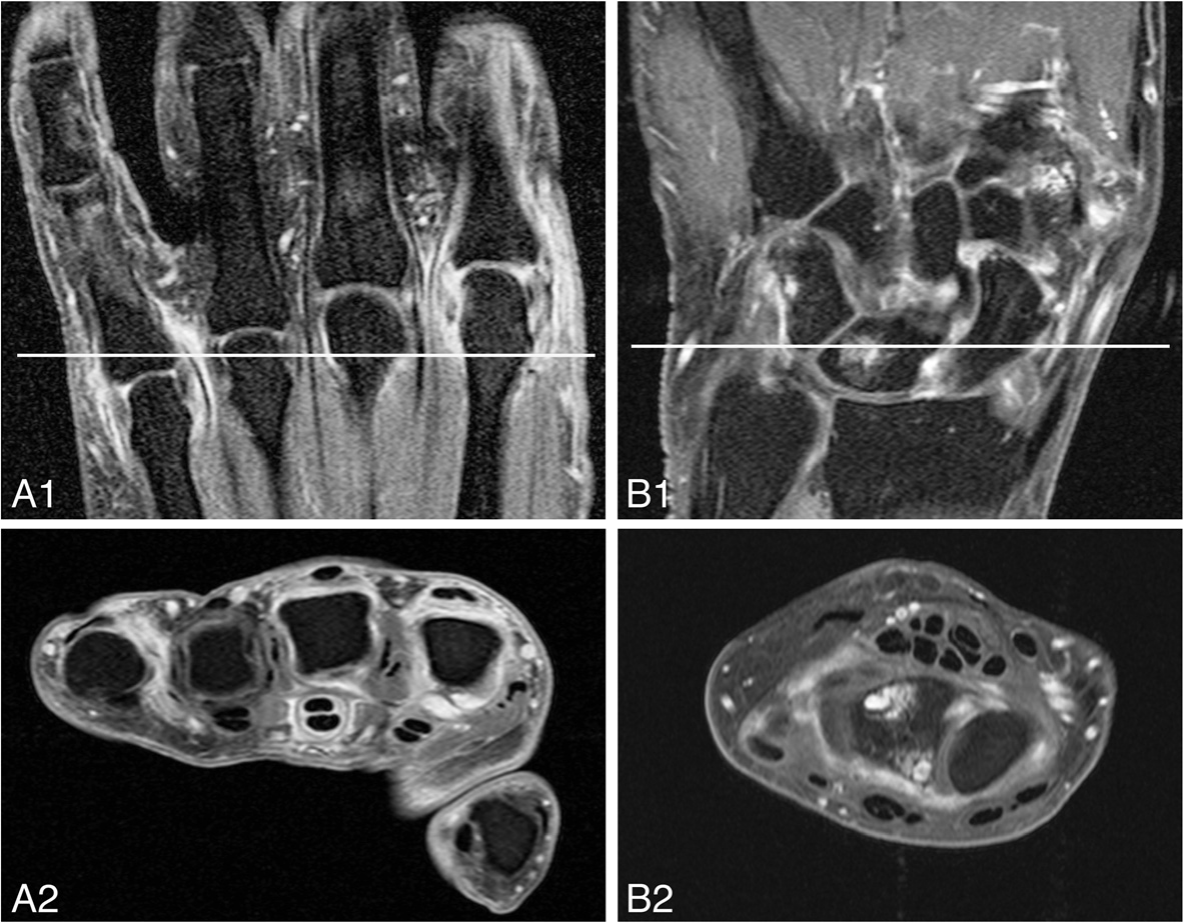
**Subclinical inflammation visualised by magnetic resonance imaging of two different anticitrullinated peptide antibody–negative arthralgia patients without clinically detectable arthritis.** Images show the metacarpophalangeal (MCP) joints and wrists of anticitrullinated peptide antibody (ACPA)–negative arthralgia patients without clinically detectable arthritis. The white lines in the top coronal images reflect the localisation of the bottom axial images. **(A)** Post–contrast enhancement coronal (A1) and axial (A2) T1-weighted fast spin echo (FSE) images with fat saturation showing enhancement of the MCP2, MCP3 and MCP5 joints, which is consistent with active synovitis. Also, pronounced tenosynovitis in the third flexor tendon is present, although tenosynovitis is not included in the OMERACT rheumatoid arthritis magnetic resonance imaging scoring system score and was not evaluated in the present study. This patient developed clinically detectable arthritis during follow-up. **(B)** Post–contrast enhancement coronal (B1) and axial (B2) T1-weighted FSE images with fat saturation showing bone marrow oedema (BME) and erosions (confirmed on the pre–contrast enhancement T1-weighted FSE sequence) in the lunate. Also, there is active synovitis in the intercarpal joint.

The proportion of patients with any sign of inflammation (synovitis and/or BME) on MRI in the wrist, MCP joints and MTP joints were, respectively, 53.1%, 20.3% and 31.3%. Synovitis was observed predominantly in the intercarpal (29.7%), radiocarpal (21.9%), MTP1 (17.2%) and MCP3 joints (14.1%). BME was most often present in the capitate (20.3%), lunate (15.6%) and MTP1 joints (15.6%). Figure 2 shows examples of inflammation visualised on MRI scans.

### Evaluation of rheumatoid factor in ACPA-negative patients

Subsequently, the ACPA-negative arthralgia patients were assigned to RF-positive (*n* = 9) and RF-negative (*n* = 55) groups. No differences in MRI inflammation, synovitis and BME scores were observed (*P* = 0.63, 0.62 and 0.90, respectively) (Additional file 1: Figure S1). We observed similar differences when the ACPA-negative RA patients were stratified.

### Evaluation of traditional measures of inflammation in ACPA-negative arthralgia patients

Furthermore, we evaluated whether the degree of inflammation visualised on MRI scans of ACPA-negative arthralgia patients was associated with the level of serological inflammation as measured by CRP levels. The synovitis score was significantly associated with CRP level (β = 0.10, *P* = 0.007), indicating that each 1 mg/L increase in CRP level resulted in a 0.10 increase in synovitis score. The BME score was not associated with CRP level (*P* = 0.88). Also, the MRI-based inflammation score was not significantly associated with CRP level (β = 0.10, *P* = 0.066).

Subsequently, we studied whether tender joints had higher MRI-based scores than nontender joints. The presence of joint tenderness was significantly associated with synovitis score (*P* = 0.026, OR = 1.15), indicating that tender joints had 15% higher odds on one-point increase in synovitis score compared to nontender joints. No significant associations were observed between joint tenderness and BME scores (*P* = 0.18) or MRI-based inflammation scores (*P* = 0.53).

### Follow-up of ACPA-negative arthralgia patients

The follow-up duration of the ACPA-negative arthralgia patients was still limited at a median of 9 months (IQR = 5 to 11). During this period, five of the ACPA-negative arthralgia patients developed clinical arthritis as detected by their rheumatologists during physical examinations (7.8%). Median (IQR) scores for MRI-based inflammation, synovitis and BME for these patients were, respectively, 5 (4 to 8.5), 3 (1.5 to 7) and 2 (1.5 to 2.5) (Figure 1). These scores were significantly higher than those of the ACPA-negative arthralgia patients who did not or had not yet developed clinical arthritis (inflammation: *P* = 0.001; synovitis: *P* = 0.002; and BME: *P* = 0.003). Of the five patients who developed clinical arthritis, three were diagnosed with RA, one with unclassified arthritis and one with psoriatic arthritis. At the time of clinical arthritis development, all patients were still ACPA-negative.

## Discussion

Early intervention in RA is associated with a more favourable disease course [1,2]. The recognition that systemic inflammatory markers are increased in the preclinical phase [6,7] and that inflammation is also locally present in small joints has increased interest in investigation of the preclinical phase of RA [8-10]. The ultimate hope is that intervention in the preclinical phase will prevent the development of the classical picture of RA. The large majority of studies on the preclinical phase have focused on patients with ACPA [3]. To the best of our knowledge, this study is the first to assess whether local subclinical inflammation is also present in ACPA-negative pre-RA patients. We observed that ACPA-negative arthralgia patients had higher MRI-based inflammation scores than healthy participants and that higher MRI-based synovitis scores were associated with higher CRP levels.

Identifying ACPA-negative arthralgia patients with an increased chance of developing RA is more challenging compared to other pre-RA studies were the presence of RA-related autoantibodies was measured and considered as a marker of increased risk. In the present study, rheumatologists were asked to select patients who, in their view, had an increased chance of developing RA. Because no type of arthralgia has yet been defined to be specific for pre-RA, we could not assign more specific criteria with regard to the type of arthralgia to be included. Retrospectively, the reasons for rheumatologists to consider patients as having an increased chance for developing RA were mainly joint pain that was worst in the early morning and improved with movement during the day (an inflammatory type of pain), the presence of morning stiffness of ≥60 minutes and a positive family history for RA. An advantage of the approach used in present study is that it resembles current clinical practice. It is of note that the studied arthralgia patients were selected from a total number of 1,335 arthralgia patients who visited our outpatient clinic between April 2012 and June 2013. The observation that 69% of the patients who were considered to have an increased chance of developing RA had any signs of subclinical inflammation on MRI scans might indicate that the rheumatologists did reasonably well in selecting ACPA-negative arthralgia patients.

The MRI inflammation scores were higher in ACPA-negative arthralgia patients than in symptom-free controls. Patients with ACPA-negative RA had much higher MRI-based inflammation scores than those in the other two groups, which was expected because these patients had clinically detectable joint inflammation. The inflammatory lesions observed in ACPA-negative arthralgia patients were small, but were located at locations that are known to be affected in RA, such as the intercarpal bones and the MCP3 and MTP1 joints [14].

Interestingly, MRI-based synovitis scores, but not BME scores, were increased in ACPA-negative arthralgia patients compared to symptom-free controls. BME is more prevalent in ACPA-positive RA patients than in ACPA-negative RA patients, and it is a strong predictor of progression of joint destruction [13,14,17]. The finding of no increase in BME score in the preclinical phase of ACPA-negative patients might suggest that BME is not an early phenomenon in ACPA-negative RA or reflects a lower prevalence of BME in ACPA-negative RA patients, a subset of RA that is also characterized by less severe radiological progression [18]. Larger and longitudinal studies are required to determine the value of BME in this disease subset.

This study has several limitations. The number of symptom-free controls studied is relatively low. Second, for ethical reasons, the controls did not receive intravenous contrast fluid. Researchers in previous studies have suggested that eliminating contrast enhancement does not affect BME scores, although it may affect the reliability of synovitis scoring [19,20]. In studies in which MRI scans with contrast enhancement were used as the gold standard, the sensitivity for synovitis scoring on the basis of high-field MRI without contrast enhancement has been reported to be high (78% to 90%), but the specificity has been reported to be moderate (31% to 79%) [19,20]. As a consequence of the moderate specificity in this study, the scores of the symptom-free controls might have been overestimated. Consequently, the differences in synovitis scores between the arthralgia patients and the healthy controls might have been underestimated. So, although the absence of contrast enhancement in the controls is a clear limitation, the results of previous studies [19,20] indicate that the differences might have been larger in cases of contrast administration to controls. Another limitation is the short duration of follow-up, which ranged from 1 to 16 months. The present study therefore provides mainly cross-sectional data. Longer follow-up is required to determine which ACPA-negative arthralgia patients and which inflammatory lesions detected by MRI are most predictive of progression to clinically detectable arthritis. Nonetheless, it is notable that arthralgia patients who developed clinical arthritis had higher MRI-based inflammation scores. A research question that remains unanswered is the long-term course of inflammation detected on MRI scans. Serial MRI scans are needed to determine whether MRI-based inflammation is relapsing, remitting or stable over time.

## Conclusions

ACPA-negative arthralgia patients, especially patients whose conditions progress to clinical arthritis, have subclinical inflammation visualised on MRI scans of the hand and foot, suggesting that also ACPA-negative RA has a preclinical symptomatic phase.

## Abbreviations

ACPA: Anticitrullinated peptide antibody; BME: Bone marrow oedema; CRP: C-reactive protein; FSE: Fast spin echo; IQR: Interquartile range; MCP: Metacarpophalangeal; MRI: Magnetic resonance imaging; MTP: Metatarsophalangeal; NSAID: Nonsteroidal anti-inflammatory drug; OMERACT: Outcome Measures in Rheumatology; PET: Positron emission tomography; PIP: Proximal interphalangeal; RA: Rheumatoid arthritis; RAMRIS: OMERACT rheumatoid arthritis magnetic resonance imaging scoring system; RF: Rheumatoid factor.

## Competing interests

The authors declare that they have no competing interests.

## Authors’ contributions

HWvS, JABvN, TWJH, MR and AHMvdHvM contributed to the conception and design of the study. HWvS and JABvN acquired and analysed the data. All authors contributed to the interpretation of the data. HWvS and AHMvdHvM wrote the first version of the manuscript, and all other authors revised it critically for important intellectual content. All authors read and approved the final manuscript.

## Supplementary Material

Additional file 1: Figure S1 Magnetic resonance imaging–based inflammation scores. **(A)** Scores for synovitis plus bone marrow edema. **(B)** Scores for synovitis. **(C)** Scores for bone marrow edema (BME). Scores are given separately for the anticitrullinated peptide antibody (ACPA)–negative patients with or without rheumatoid factor (RF). The scores of all participants are presented individually (dots) and as the median scores per group (horizontal line). The *y*-axes are split because the RA patients had higher scores than the ACPA-negative arthralgia patients. The presented *P*-values were obtained by comparing the scores of the RF-negative and RF-positive patients within the group of ACPA-negative arthralgia and ACPA-negative RA patients. Click here for file
