# Supplementary figures and images for: Subclinical inflammation on MRI of hand and foot of anticitrullinated peptide antibody–negative arthralgia patients at risk for rheumatoid arthritis
Source: Arthritis Res Ther. 2014 Apr 10;16(2):R92. doi: 10.1186/ar4536 (PMC4060237; doi:10.1186/ar4536)

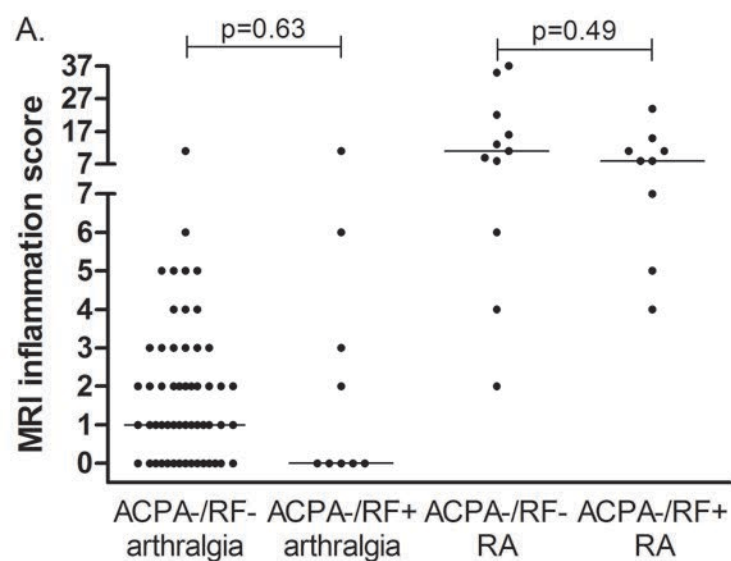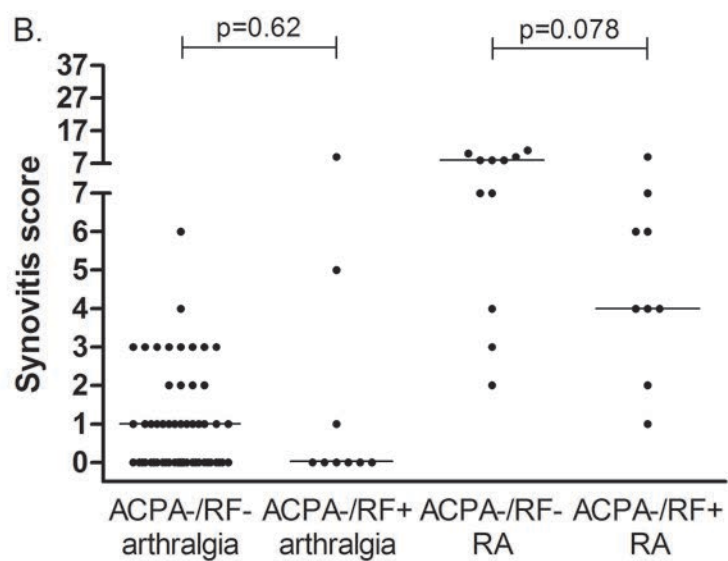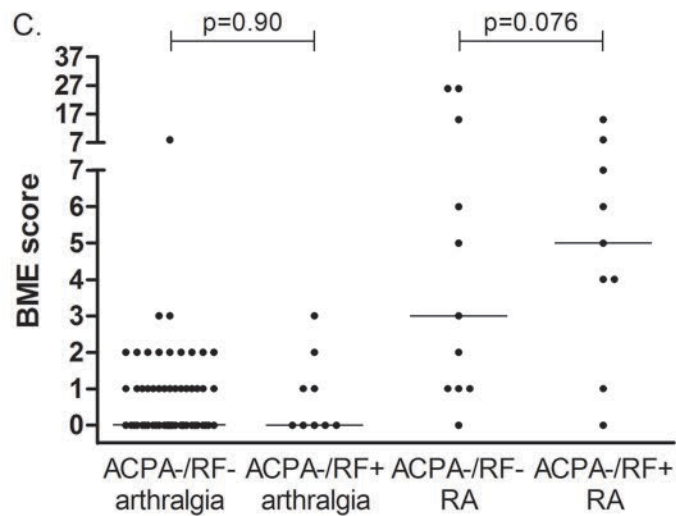

Supplement: Additional file 1: Figure S1 — Magnetic resonance imaging–based inflammation scores. (A) Scores for synovitis plus bone marrow edema. (B) Scores for synovitis. (C) Scores for bone marrow edema (BME). Scores are given separately for the anticitrullinated peptide antibody (ACPA)–negative patients with or without rheumatoid factor (RF). The scores of all participants are presented individually (dots) and as the median scores per group (horizontal line). The y-axes are split because the RA patients had higher scores than the ACPA-negative arthralgia patients. The presented P-values were obtained by comparing the scores of the RF-negative and RF-positive patients within the group of ACPA-negative arthralgia and ACPA-negative RA patients. [file ar4536-S1.pdf]
